# Early-life and adult body mass index in relation to MS disability progression

**DOI:** 10.1007/s00415-025-13319-2

**Published:** 2025-08-12

**Authors:** Lars Alfredsson, Jie Guo, Eva Johansson, Tomas Olsson, Anna Karin Hedström

**Affiliations:** 1 Institute of Environmental Medicine, Karolinska Institutet, Stockholm, Sweden; 2 Centre for Occupational and Environmental Medicine, Region Stockholm, Stockholm, Sweden; 3 Department of Nutrition and Health, China Agricultural University, Beijing, China; 4 Department of Clinical Neuroscience, Karolinska Institutet, Visionsvägen 18, L8, 171 76 Stockholm, Sweden

**Keywords:** Multiple sclerosis, Body mass index, Obesity, Weight loss, Disability worsening, Disability progression, Expanded disability status scale

## Abstract

**Background and objectives:**

The influence of body weight across the life course on multiple sclerosis (MS) progression remains incompletely understood. While excess body mass at diagnosis is associated with disability progression, it is unclear how early-life and adult BMI jointly affect long-term outcomes. We aimed to investigate the separate and combined effects of BMI at age 20 and at diagnosis on MS progression.

**Methods:**

We studied 2940 individuals with relapsing-onset MS from a population-based case–control study with prospective follow-up through the Swedish MS registry. BMI was calculated from self-reported weight at age 20 and at diagnosis. Outcomes included confirmed disability worsening (CDW), and time to reach EDSS 3 and EDSS 4. Cox regression and general linear models were used to examine associations between BMI and MS progression, including interaction terms.

**Results:**

High BMI (> 28 kg/m^2^) at age 20 was associated with higher disability at diagnosis (β = 0.15, *p* = 0.0015), while BMI at diagnosis predicted increased risk of progression. Compared to individuals with BMI ≤ 28 kg/m^2^ at both time points, those with persistent elevated BMI had higher risks of CDW (HR 1.28, 95% CI 1.01–1.63), EDSS 3 (HR 1.64, 95% CI 1.21–2.24), and EDSS 4 (HR 1.51, 95% CI 1.00–2.39). Risks were increased, though less pronounced, among those with high BMI only at diagnosis. Early-life excess weight alone was not associated with progression. Interaction models suggested a stronger effect of adult BMI in the presence of early excess weight.

**Conclusions:**

High BMI at diagnosis was associated with faster disability progression, particularly when present since early adulthood. These findings underscore the potential benefits of early weight management in MS.

## Background

Multiple sclerosis (MS) is a chronic, immune-mediated disorder of the central nervous system, characterized by demyelination, axonal injury, and progressive neurodegeneration. Its etiology is believed to reflect a complex interplay between genetic susceptibility and environmental risk factors [1]. Obesity has emerged as a significant risk factor in MS, not only influencing disease susceptibility [2–4] but also shaping long-term prognosis [5, 6]. Several studies have shown that higher body mass index (BMI) at the time of diagnosis is associated with faster disability progression [5, 6], suggesting that adiposity may influence disease activity and neurodegeneration beyond the initial disease onset. Proposed mechanisms include chronic systemic inflammation, adipokine signaling, metabolic dysfunction, and altered immune regulation [7, 8], all of which may contribute to accelerated central nervous system damage in MS.

However, studies examining the impact of weight loss after diagnosis among individuals who were obese at the time of diagnosis have not demonstrated a clearly favorable effect on disease progression. In prior work, individuals who were obese at diagnosis and subsequently lost weight and were no longer classified as obese did not show improved outcomes compared to those who remained obese [6]. Similarly, in a study of individuals with MS who underwent bariatric surgery, no apparent benefit in terms of EDSS progression was observed compared to matched obese individuals with MS who did not undergo surgery [9]. These findings raise the possibility that the long-term consequences of adiposity may be established earlier in life and that weight reduction later in adulthood may be insufficient to modify disease trajectory.

Using a cohort of newly diagnosed individuals with MS, we aimed to assess whether early-life obesity exerts a lasting influence on disease outcomes, and whether outcomes differ between individuals who lost or gained weight between early adulthood and diagnosis.

## Methods

The study comprises cases from the Epidemiologic Investigation of Multiple Sclerosis (EIMS), a Swedish case–control study enrolling individuals with incident MS. Cases were recruited from hospital-based neurology units, including all university hospitals in Sweden, between April 2005 and December 2019, yielding a total of 3567 participants. The response rate among cases was 93%. All diagnoses were made by neurologists in accordance with the McDonald criteria [10, 11]. Additional details regarding study design and methodology have been described previously [12].

Of the 3567 cases, 3365 (94%) had available follow-up data on Expanded Disability Status Scale (EDSS) scores in the Swedish MS registry [13]. We excluded those with BMI at diagnosis below 18.5 kg/m^2^ due to potential underlying comorbidities (*n* = 116), those younger than 20 years at diagnosis (*n* = 189), and those with progressive-onset disease course (*n* = 120). The final analytical sample included 2940 individuals with relapsing-onset MS. The study was approved by the Regional Ethical Review Board at Karolinska Institute and has been performed in accordance with the ethical standards laid down in the 1964 Declaration of Helsinki and its later amendments.

### Definition of exposures

We used a standardized questionnaire to collect data on environmental exposures and lifestyle habits. BMI at age 20 and at diagnosis was calculated from self-reported height and weight. We assess changes in adiposity over time; we initially grouped participants into three BMI categories: < 25 kg/m^2^, 25–28, and > 28 kg/m^2^. For the main analysis, we used a binary classification based on a cutoff of 28 kg/m^2^ to distinguish between lower and higher adiposity at both time points. Although the World Health Organization defines obesity as BMI > 30 kg/m^2^, few individuals in our cohort transitioned from obesity in early adulthood to non-obese status at diagnosis (1.6%). To ensure sufficient group sizes and enable meaningful analysis of weight change, we used 28 kg/m^2^ as a pragmatic threshold, representing the upper range of overweight and the transition toward obesity.

### Outcome measures

The Swedish MS registry is used nationwide across all neurology units and includes prospectively recorded data entered by physicians and nurses on clinical measures, disease-modifying treatments, and patient-related outcomes [14]. Baseline was defined as the date of the first recorded EDSS score (mean time between inclusion in EIMS and baseline EDSS assessment was 0.02 years, SD = 2.3). Confirmed disability worsening (CDW) was defined as an increase in EDSS by at least 1 point from baseline, sustained for a minimum of six months, with adjustments based on baseline EDSS (≥ 1.5 points if EDSS was 0, or ≥ 0.5 points if EDSS was > 5.5). Time to reaching EDSS 3 and 4 was analyzed as secondary outcomes, restricted to participants with a baseline EDSS < 3. To complement EDSS-based outcomes, we also examined patient-reported physical and psychological worsening using the physical and psychological components of the Multiple Sclerosis Impact Scale 29 (MSIS-29). A clinically meaningful worsening was defined as an increase of 7.5 points or more.

### Statistical analysis

Categorical variables were summarized using frequencies and percentages, and continuous variables using means and standard deviations (SD). To assess potential multicollinearity between BMI at age 20 and BMI at diagnosis, we examined the Pearson correlation and calculated the variance inflation factor (VIF). We assessed the joint association of BMI at age 20 and at MS diagnosis with the risk of disability progression using Cox proportional hazards regression. Participants with BMI < 28 kg/m^2^ at both time points served as the reference group. Follow-up time was calculated from baseline, defined as the first recorded EDSS score, to the occurrence of each endpoint, death, or end of follow-up. The proportional hazard assumption was evaluated using Schoenfeld residuals and was met for all models.

We used general linear models to assess whether BMI at age 20 or at diagnosis was associated with baseline EDSS score. To examine whether the effect of BMI at diagnosis on disability progression varied by early-life BMI status, both BMI measures were modeled as continuous variables together with an interaction term. Interaction models were visualized by plotting adjusted hazard ratios and 95% confidence intervals across a range of BMI values, evaluated at fixed levels of the interacting BMI variable.

All models were adjusted for age at diagnosis, sex, disease duration, baseline EDSS, smoking status, and disease-modifying therapy. Smoking was categorized as never, past, or current at diagnosis. Disease-modifying treatment was accounted for by calculating the proportion of follow-up time on low- and high-efficacy treatments.

Physical activity and sun exposure were included in sensitivity analyses. Physical activity was categorized into four levels based on leisure-time exercise frequency (sedentary leisure time, moderate exercise, regular exercise 1–2 times/week, and regular exercise 3 or more times/week). Sun exposure was indexed from three questionnaire items (sunbathing, traveling to sunny locations, and sunbed use) and dichotomized at the median. As these adjustments had negligible influence on the results, they were not included in the final models. Sensitivity analyses were conducted using alternative BMI cutoffs of 27.5 kg/m^2^ and 28.5 kg/m^2^ for defining adiposity status. Finally, Kaplan–Meier curves were used to illustrate the unadjusted time to CDW, EDSS 3, and EDSS 4 across the four BMI groups. All analyses were conducted in SAS version 9.4 (SAS Institute, Cary, NC, USA) and figures were created in Python version 3.11.9 using Matplotlib.

## Results

We followed up 2940 individuals with incident MS and available data on BMI at age 20 and at diagnosis. Mean age at baseline was 37.6 years, and mean duration since the onset of disease was 2.5 years. Baseline characteristics of cases, overall and by joint BMI status, are presented in Table 1.

**Table 1:** Baseline characteristics of overall sample and by BMI status at diagnosis and at age 20 years

	Total	BMI at diagnosis < 28		BMI at diagnosis ≥ 28	
BMI at age 20 years	22.8 (4.0)	< 28	> 28	< 28	> 28
N	2940	2302	58	420	160
Age at diagnosis (SD)	37.6 (10.5)	37.4 (10.6)	31.7 (7.3)	41.3 (8.3)	32.9 (8.9)
Female, n (%)	2095 (71.3)	1626 (70.6)	40 (69.0)	308 (73.3)	121 (75.6)
Nordic origin, n (%)	2330 (79.9)	1818 (79.6)	47 (82.5)	341 (82.2)	124 (78.5)
Disease duration at diagnosis, years (SD)	2.5 (3.7)	2.6 (3.8)	1.4 (2.4)	2.5 (3.7)	2.2 (3.8)
No treatment, n (%)	217 (7.4)	167 (7.3)	1 (1.7)	37 (8.8)	12 (7.5)
Low-efficacy treatment, n (%)	957 (32.5)	740 (32.2)	20 (34.5)	153 (36.4)	44 (27.5)
High-efficacy treatment, n (%)	1766 (60.1)	1395 (60.6)	37 (63.8)	230 (54.8)	104 (65.0)
Time from diagnosis to treatment start, years (SD)	0.3 (1.5)	0.3 (1.4)	0.04 (0.3)	0.4 (1.9)	0.2 (0.9)
Baseline EDSS (SD)	1.7 (1.4)	1.7 (1.3)	1.5 (1.2)	1.8 (1.5)	1.9 (1.4)
Current smoking, n (%)	656 (22.3)	491 (21.3)	20 (34.5)	96 (22.9)	49 (30.6)
Pack years of smoking (SD)	8.5 (16.0)	8.8 (18.0)	4.5 (3.8)	8.5 (7.2)	8.0 (9.2)
Sun exposure index (SD)	6.2 (1.8)	6.3 (1.8)	6.0 (1.9)	5.8 (1.8)	5.6 (1.7)
Alcohol, n (%)	1999 (68.0)	1605 (69.7)	35 (60.3)	271 (64.5)	88 (55.0)
Alcohol (gram/week, SD)	44.7 (90.1)	43.3 (69.0)	43.3 (69.0)	44.7 (71.3)	39.4 (75.0)
Physical activity index (SD)	2.4 (1.0)	2.4 (0.9)	2.6 (1.0)	2.0 (0.9)	2.1 (0.9)

There was low collinearity between BMI at age 20 and BMI at diagnosis (Pearson *r* = 0.11; R^2^ = 0.0015; VIF≈1.00). The distribution of participants across BMI categories at age 20 and at diagnosis is shown in Table 2. The majority (54.0%) had BMI < 28 kg/m^2^ at both time points. A smaller proportion (5.4%) had persistently elevated BMI, while 14.3% had elevated BMI only at diagnosis. Few participants (2.0%) had elevated BMI only at age 20.

**Table 2:** Relationship between BMI at age 20 and at time of MS diagnosis

BMI at age 20	BMI at diagnosis		
	< 25 (*n* = 1683)	25–28 (*n* = 677)	> 28 (*n* = 580)
< 25, n (%)	1588 (67.1)	520 (22.0)	259 (10.9)
25–28, n (%)	78 (22.0)	116 (32.7)	161 (45.4)
> 28, n (%)	17 (7.8)	41 (18.8)	160 (73.4)

Higher BMI at age 20 was significantly associated with greater disability at diagnosis (β = 0.15, p = 0.0015), whereas BMI at diagnosis showed no significant association with baseline EDSS (β = –0.01, p = 0.83).

**Fig. 1:**
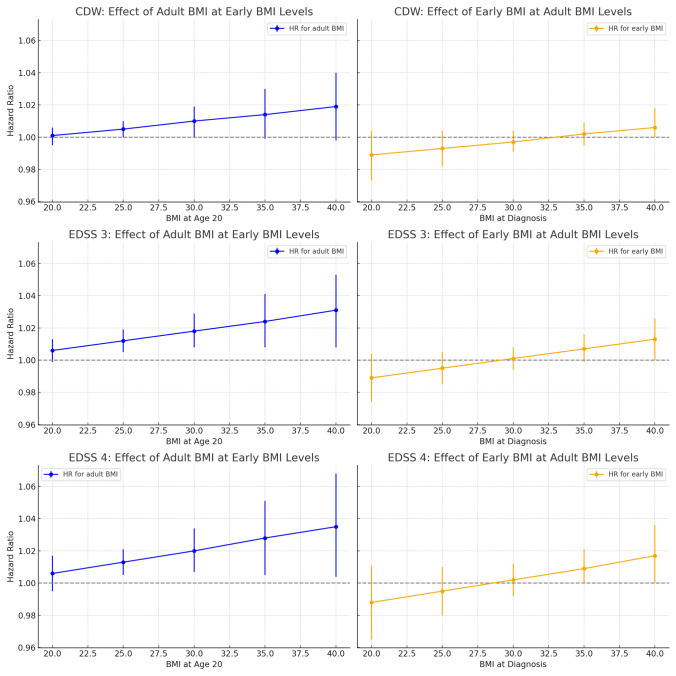
Interaction between early-life and adult BMI in relation to MS progression outcomes. Each panel presents the estimated effect of one BMI measure (adult or early) across fixed levels of the other, based on Cox proportional hazards models with an interaction term between continuous BMI at age 20 and BMI at diagnosis. Left column: hazard ratios for increasing adult BMI at fixed early-life BMI levels. Right column: hazard ratios for increasing early-life BMI at fixed adult BMI levels. Outcomes include confirmed disability worsening (CDW, top row), time to EDSS 3 (middle row), and time to EDSS 4 (bottom row). HR > 1 indicates increased risk of progression. Models were adjusted for age at diagnosis, sex, disease duration, baseline EDSS, disease-modifying therapy, and smoking. Error bars represent 95% confidence intervals

Compared to individuals with BMI < 28 kg/m^2^ at both time points, those with BMI > 28 kg/m^2^ at diagnosis had higher risks of unfavorable outcomes, regardless of earlier BMI status (Table 3). Those with persistent elevated BMI had higher risks of CDW (HR 1.28, 95% CI 1.01–1.63), EDSS 3 (HR 1.64, 95% CI 1.21–2.24), and EDSS 4 (HR 1.51, 95% CI 1.00–2.39). Risks were increased, though less pronounced, among those with high BMI only at diagnosis. In contrast, those with elevated BMI at age 20 but not at diagnosis did not show increased risk for any outcome. Similar results were observed when using patient-reported physical and psychological worsening, defined as an increase of ≥ 7.5 points in the MSIS-29 physical and psychological score, respectively. Individuals with high BMI at diagnosis had increased risk of physical worsening (HR 1.20, 95% CI 1.01–1.43, for elevated BMI only at diagnosis and HR 1.39, 95% CI 1.06–1.80, for persistently elevated BMI), while those with high early-life BMI only did not differ significantly from the reference group (Table 3). The risk of EDSS progression increased with higher BMI at diagnosis, particularly among individuals who also had high BMI at age 20. For example, among those with a BMI of 30 at age 20, each unit increase in BMI at diagnosis was associated with a higher risk of reaching EDSS 3 (HR 1.018, 95% CI 1.008–1.029) and EDSS 4 (HR 1.020, 95% CI 1.007–1.034). Although the association between BMI at diagnosis and progression was somewhat attenuated among those with low early BMI, the direction of effect remained consistent across all early BMI levels. Conversely, BMI at age 20 had limited influence among those with low BMI at diagnosis, but risk estimates increased with higher adult BMI (Table 4, Fig. 1).

**Table 3:** HR with 95% CI of having unfavorable outcomes post-diagnosis, by BMI status at diagnosis and at age 20

First clinical disease worsening (CDW)						
BMI at diagnosis	BMI at age 20	N	Years (SD)	Outcome (%)	HR (95% CI)^a^	HR (95% CI)^b^
< 28	< 28	2302	6.1 (4.4)	1039 (45.1)	1.0 (reference)	1.0 (reference)
< 28	> 28	58	6.1 (4.3)	22 (37.9)	0.93 (0.61–1.41)	0.94 (0.60–1.40)
> 28	< 28	420	5.6 (3.8)	197 (46.9)	1.05 (0.90–1.23)	1.08 (0.93–1.26)
> 28	> 28	160	5.1 (4.1)	69 (43.1)	1.21 (0.97–1.55)	1.28 (1.01–1.63)

EDSS 3						
BMI at diagnosis	BMI at age 20	N	Years (SD)	Outcome (%)	HR (95% CI)^a^	HR (95% CI)^b^
< 28	< 28	1874	7.3 (4.7)	524 (28.0)	1.0 (reference)	1.0 (reference)
< 28	> 28	48	7.2 (4.5)	12 (25.0)	1.05 (0.59–1.86)	1.06 (0.59–1.88)
> 28	< 28	323	6.5 (4.2)	101 (31.3)	1.16 (0.95–1.43)	1.19 (1.01–1.46)
> 28	> 28	122	6.5 (4.2)	44 (36.1)	1.83 (1.34–2.50)	1.64 (1.21–2.24)

EDSS 4						
BMI at diagnosis	BMI at age 20	N	Years (SD)	Outcome (%)	HR (95% CI)^a^	HR (95% CI)^b^
< 28	< 28	1874	8.2 (4.7)	220 (11.7)	1.0 (reference)	1.0 (reference)
< 28	> 28	48	8.0 (4.7)	4 (8.3)	0.90 (0.43–2.41)	0.90 (0.43–2.43)
> 28	< 28	323	7.5 (4.2)	51 (15.8)	1.35 (1.00–1.83)	1.44 (1.06–1.95)
> 28	> 28	122	7.0 (4.6)	19 (15.6)	1.81 (1.13–2.90)	1.51 (1.00–2.39)

Physical worsening (increased MSIS-29 physical score by 7.5 or more)						
BMI at diagnosis	BMI at age 20	N	Years (SD)	Outcome (%)	HR (95% CI)^a^	HR (95% CI)^b^^,c^
< 28	< 28	1854	5.0 (4.1)	736 (39.7)	1.0 (reference)	1.0 (reference)
< 28	> 28	54	5.4 (3.8)	22 (40.7)	0.96 (0.63–1.47)	1.04 (0.69–1.59)
> 28	< 28	334	4.6 (4.8)	150 (44.9)	1.23 (1.06–1.51)	1.20 (1.01–1.43)
> 28	> 28	134	4.8 (5.7)	61 (45.5)	1.27 (0.97–1.62)	1.39 (1.06–1.80)

Psychological worsening (increased MSIS-29 physical score by 7.5 or more)						
BMI at diagnosis	BMI at age 20	N	Years (SD)	Outcome (%)	HR (95% CI)^a^	HR (95% CI)^b^^,d^
< 28	< 28	1854	4.6 (4.1)	910 (49.1)	1.0 (reference)	1.0 (reference)
< 28	> 28	54	4.9 (3.9)	27 (50.0)	0.99 (0.67–1.45)	1.04 (0.71–1.53)
> 28	< 28	334	4.3 (4.5)	174 (52.1)	1.16 (0.98–1.36)	1.25 (1.06–1.47)
> 28	> 28	134	4.2 (5.5)	76 (56.7)	1.22 (0.94–1.57)	1.28 (1.00–1.66)

^a^crude

^b^adjusted for age at diagnosis, sex, disease duration, baseline EDSS, disease-modifying therapy, and smoking

^c^adjusted for baseline MSIS-29 physical score

^d^adjusted for baseline MSIS-29 psychological score

**Table 4:** HR per unit increase in BMI for EDSS progression outcomes associated with BMI at diagnosis and at age 20, from Cox models with interaction terms

	CDW	EDSS 3	EDSS 4
BMI at diagnosis	HR (95% CI)	HR (95% CI)	HR (95% CI)
at BMI = 20 at age 20	1.001 (0.995–1.006)	1.006 (0.999–1.013)	1.006 (0.996–1.017)
at BMI = 25 at age 20	1.005 (1.000–1.010)	1.012 (1.005–1.019)	1.013 (1.005–1.021)
at BMI = 30 at age 20	1.010 (1.000–1.019)	1.018 (1.008–1.029)	1.020 (1.007–1.034)
at BMI = 35 at age 20	1.014 (1.000–1.030)	1.024 (1.008–1.041)	1.028 (1.005–1.051)
at BMI = 40 at age 20	1.019 (1.000–1.040)	1.031 (1.008–1.053)	1.035 (1.004–1.068)
BMI at 20 years	HR (95% CI)	HR (95% CI)	HR (95% CI)
at BMI = 20 at diagnosis	0.989 (0.973–1.004)	0.989 (0.976–1.004)	0.990 (0.969–1.011)
at BMI = 25 at diagnosis	0.993 (0.982–1.004)	0.995 (0.989–1.005)	0.995 (0.980–1.010)
at BMI = 30 at diagnosis	0.997 (0.992–1.005)	1.001 (0.996–1.008)	1.002 (0.996–1.012)
at BMI = 35 at diagnosis	1.002 (0.998–1.010)	1.007 (1.000–1.016)	1.009 (1.000–1.021)
at BMI = 40 at diagnosis	1.006 (1.000–1.018)	1.013 (1.000–1.026)	1.017 (1.000–1.036)

Estimates represent adjusted HRs with 95% CI for each unit increase in BMI at either diagnosis or age 20, evaluated at specific levels of the other BMI variable. Models are adjusted for age at diagnosis, sex, disease duration, baseline EDSS, disease-modifying therapy, and smoking; BMI = body mass index; HR = hazard ratio; CDW = confirmed disability worsening

Sensitivity analyses using alternative BMI cutoffs of 27.5 kg/m^2^ and 28.5 kg/m^2^ yielded similar results, supporting the robustness of the findings. The unadjusted Kaplan–Meier curves illustrate similar patterns as observed in the adjusted Cox models, with shorter event-free time among those with high BMI at diagnosis, regardless of earlier BMI status (eFigures S1–S3). In contrast, individuals with elevated BMI in early adulthood but normal weight at diagnosis did not show worse outcomes, suggesting that current rather than past adiposity is more strongly associated with MS progression.

## Discussion

In this cohort of individuals with incident relapsing-onset MS, current adiposity at diagnosis remained the primary predictor of unfavorable outcomes, while early-life adiposity may play a modifying role. Our results support a joint and possibly cumulative effect of high BMI across the life course on MS progression.

Elevated BMI at diagnosis was more commonly due to weight gain after early adulthood rather than persistent obesity from a young age. Only a small subgroup had elevated BMI exclusively in early adulthood, reflecting the rarity of sustained weight loss from obesity.

Early-life adiposity was associated with greater neurological impairment at diagnosis, as indicated by higher baseline EDSS scores. This may reflect long-term systemic effects of adiposity present before the onset of clinically overt MS and suggests that adiposity in early adulthood might contribute to preclinical disease burden. Potential mechanisms include early-life metabolic imprinting through adipokine signaling, low-grade systemic inflammation, or immune dysregulation that persists even after weight normalization, potentially contributing to subclinical neuroinflammation or priming of the immune system prior to disease onset. In contrast, BMI at diagnosis showed no such association, raising the possibility that metabolic exposures in early adulthood may influence disease severity even before clinical diagnosis.

Individuals with elevated BMI at age 20 were, on average, younger at the time of MS diagnosis than those with lower early-life BMI. This observation suggests that early-life adiposity may influence not only disease severity at onset but also the timing of clinical manifestation. Although the underlying mechanisms remain speculative, it raises the possibility that systemic inflammation or metabolic dysfunction present in early adulthood may accelerate subclinical disease processes and advance the onset of clinically overt MS.

When evaluating long-term outcomes, individuals with high BMI at diagnosis, regardless of earlier BMI status, had a higher risk of confirmed disability worsening and reaching EDSS milestones. The highest risks were observed among individuals with persistently high BMI from early adulthood into the time of diagnosis, indicating a potential cumulative impact of sustained adiposity. However, individuals with elevated BMI in early adulthood who had lost weight by the time of diagnosis did not show increased risk of progression. This indicates that the association between early BMI and baseline disability does not necessarily translate into worse long-term prognosis, and that later improvements in body weight may mitigate further negative effects. Given that previous studies have not shown beneficial effects on outcomes following weight loss after diagnosis [6, 9], interventions aimed at reducing excess weight early in the disease course may be more effective than those introduced later. This has particular relevance in light of the updated McDonald criteria [14], which allow for earlier diagnosis of MS and, potentially, earlier implementation of lifestyle interventions. Timely weight management in newly diagnosed individuals could represent a window of opportunity to influence long-term outcomes more effectively.

Analyses using continuous measures confirmed that the prognostic influence of BMI at diagnosis was stronger in those with higher early BMI. While BMI at age 20 alone did not predict worse outcomes among individuals with normal adult weight, it contributed to elevated risk among those with adult obesity, supporting a cumulative effect of high BMI across the life course. While these findings suggest a cumulative effect of adiposity across the life course, it remains unclear whether this reflects the same underlying biological pathways or distinct, stage-specific processes that require ongoing excess weight to manifest.

We used a cutoff of 28 kg/m^2^ to identify individuals with elevated adiposity, a threshold that approximates the upper range of overweight and the transition toward obesity. This pragmatic approach allowed us to capture a broader range of elevated BMI while ensuring sufficient group sizes for statistical analysis. This cutoff was particularly relevant in our cohort, as only a very small proportion of participants met the WHO obesity definition at age 20 and later transitioned to a non-obese BMI by diagnosis. Thus, using a slightly lower threshold enabled a more meaningful assessment of weight change across time. The consistency of findings across both categorical and continuous BMI analyses strengthens the robustness of our conclusions. Moreover, sensitivity analyses using alternative cutoffs of 27.5 kg/m^2^ and 28.5 kg/m^2^ yielded similar results, further supporting the stability of the observed associations.

Several limitations of this study should be acknowledged. Although BMI is a widely used indicator of adiposity, it does not distinguish between fat and lean mass and may not fully reflect fat distribution. Additionally, obesity may affect mobility and thereby influence EDSS scores independently of neurological progression, which could lead to misclassification of outcomes. To address this, we included the physical and psychological components of the MSIS-29 as a complementary, patient-reported measure of disability, which showed consistent results. BMI at age 20 was based on retrospective self-report, which may be subject to some degree of underreporting, particularly in individuals with higher body weight. However, previous studies have shown high correlations between self-reported and measured weight, with only minor discrepancies [15, 16]. The timing and duration of weight change could not be assessed, limiting our ability to explore dose–response or temporal relationships. While we adjusted for various covariates, residual confounding remains possible, particularly for time-varying factors not captured in the baseline-adjusted models.

In conclusion, elevated BMI at diagnosis was associated with increased risk of disability progression, particularly when excess weight had been present since early adulthood. While early-life adiposity was linked to higher disability at diagnosis, it only predicted long-term progression when high BMI persisted. Participants who reduced weight before diagnosis did not show increased risk of progression, suggesting potential benefits of weight loss before or early in the disease course.

## Data Availability

Anonymized data underlying this article will be shared on reasonable request from any qualified investigator that wants to analyze questions that are related to the published article.
